# Seroprevalence of campylobacteriosis and relevant post-infectious sequelae

**DOI:** 10.1007/s10096-013-2040-4

**Published:** 2014-01-12

**Authors:** A. E. Zautner, C. Johann, A. Strubel, C. Busse, A. M. Tareen, W. O. Masanta, R. Lugert, R. Schmidt-Ott, U. Groß

**Affiliations:** 1 Institut für Medizinische Mikrobiologie, Universitätsmedizin Göttingen, Kreuzbergring 57, 37075 Göttingen, Germany; 2 UMG-Labor, Institut für Klinische Chemie/Zentrallabor, Universitätsmedizin Göttingen, Göttingen, Germany; 3 Sekisui Virotech GmbH, Rüsselsheim, Germany; 4 Present Address: GlaxoSmithKlineVaccines, Wavre, Belgium

## Abstract

Post-infectious sequelea such as Guillain Barré syndrome (GBS), reactive arthritis (RA), and inflammatory bowel disease (IBD) may arise as a consequence of acute *Campylobacter*-enteritis (AE). However, reliable seroprevalence data of *Campylobacter-*associated sequelae has not been established. The objectives of this study were, first, to identify the most specific and sensitive test antigen in an optimized ELISA assay for diagnosing a previous *Campylobacter*-infection and, second, to compare the prevalence of anti-*Campylobacter* antibodies in cohorts of healthy blood donors (BD), AE, GBS, RA, and IBD patients with antibodies against known GBS, RA and IBD triggering pathogens. Optimized ELISAs of single and combined *Campylobacter-*proteins OMP18 and P39 as antigens were prepared and sera from AE, GBS, RA and IBD patients and BD were tested for *Campylobcter-*specific IgA and IgG antibodies. The results were compared with MIKROGEN™-recomLine *Campylobacter* IgA/IgG and whole cell lysate-immunoblot. Antibodies specific for *Helicobacter pylori*, *Mycoplasma pneumoniae*, *Yersinia enterocolitica*, and *Borrelia afzelii* were tested with commercial immunoblots. ROC plot analysis revealed AUC maxima in the combination of OMP18 and P39 for IgA and in the P39-antigen for IgG. As a result, 34–49 % GBS cases, 44–62 % RA cases and 23–40 % IBD cases were associated with *Campylobacter-*infection. These data show that *Campylobcater*-seropositivity in these patient groups is significantly higher than other triggering pathogens suggesting that it plays an important role in development of GBS and RA, and supports the hypothesis that recurrent acute campylobacteriosis triggers IBD.

## Introduction

Members of genus *Campylobacter* are Gram-negative and microaerophilic bacteria that invade the gastrointestinal tract of humans causing campylobacteriosis whose clinical symptoms include bloody or watery diarrhea, abdominal pain, fever, headache, nausea and vomiting. Although this acute enteritis is self-limiting, post-infectious sequelae GBS, RA and IBD can arise after recovery [1–3]. Recently, *C. jejuni* has been found to be the leading cause of bacterial gastroenteritis worldwide [4, 5], which has led to renewed interest in quantifying the seroprevalence of *Campylobacter*-specific antibodies in the rising cases of GBS, RA and IBD as post-infectious sequelae.

Recent studies have shown GBS an autoimmune disorder in which the body’s immune system attacks GM-gangliosides in the central nervous system leading to acute neuromuscular paralysis and consecutive muscle weakness succeeding campylobacteriosis [6]. Furthermore, cytomegaloviruses (CMV), Epstein-Barr viruses (EBV) and *Mycoplasma pneumonia* have been shown to trigger GBS [6]. Presently, four common types of GBS are recognized *vis-á-vis* the Miller Fisher syndrome (MFS), the acute motor axonal neuropathy (AMAN), the acute inflammatory demyelinating polyradiculoneuropathy (AIDP) and the acute motor-sensory axonal neuropathy (AMSAN) [6]. Importantly, *Campylobacter* has been linked to trigger MFS, AMAN, and AMSAN [6].

Similarly, RA has been shown to develop after campylobacteriosis [7–9]. Like in GBS, other pathogens including *Salmonella enterica*, *Shigella dysenteriae*, *Yersinia enterocolitica*, *Yersinia pseudotuberculosis*, and *Chlamydia trachomatis* have been implicated in triggering RA [7]. In previous studies the incidence of RA in acute campylobacteriosis patients was found to range from 1 to 7 % [7, 10]. However, seroprevalence data in RA patients, with an acute flare up of arthritis has not been estimated so far.

Equivalently, epidemiologic, ecologic and genetic studies have associated the pathogenesis of IBD, a strictly gastrointestinal tract immunological disorder, with interplay between *C. jejuni*, host genetic susceptibility, recurrent AE, and commensal microflora [11–15]. It has been revealed that host genetics influences the diversity and load of commensal microflora. However, slight alteration in diversity and loads of members of the commensal microflora of phyla Firmicutes and Bacterioidetes due to diet and other unknown agents, promotes intestinal epithelial invasion by *C. jejuni* leading to development of IBD [15–17].

The *Campylobacter* literature shows inconsistence in the frequency of previous *Campylobacter* infections in these sequelae. As a consequence, there is under- or over-estimation of *Campylobacter*-triggered post infectious sequelae. This has been attributed to lack of reliable serological assays for detecting previous *Campylobacter* infections due to poor standardization and cross-reactivity to other pathogens including *Helicobacter* spp., *Arcobacter* spp., *Salmonella* spp., *Legionella* spp., *Yersinia* spp., and *Corynebacterium* spp. [18–20].

Recently, we reported on a *Campylobacter* ELISA with 91.9 % sensitivity and 99.0 % specificity that is reliable for detecting previous *Campylobacter* antibodies in healthy individuals (BD), AE-patients and GBS-patients [21]. This assay is based on a combination of two purified *Campylobacter* antigens, namely, OMP18 and P39 [22]. However, the most specific and sensitive antigen or antigen combination for the detection of previous *Campylobacter* infection in a particular post-infectious sequel remains unknown. Furthermore, the ability of antigens OMP18 and P39 to diagnose previous *Campylobacter* infection in RA-patients and IBD-patients is also unknown. Clearly, knowledge of the specificity and sensitivity of antigens OMP18 and P39 is important for continuous development of reliable assays for detecting previous *Campylobacter* infections in a particular post-infectious sequel.

In the present study, we investigated the most specific and sensitive antigen between OMP18, P39 and combined OMP18 + P39 for detecting *Campylobacter* specific antibodies in AE-patients and patients of each named post-infectious sequel; we tested sensitivity and specificity of optimized OMP18 and P39 ELISA in detecting prior *Campylobacter* infections by comparing it’s results with those of antigens MOMP, PEB1, PEB2, PEB4, OMP18, and P39 embedded in MIKROGEN^TM^-recomLine *Campylobacter* IgA/IgG blot and of a whole cell lysate immunoblot [23]; we used the optimized OMP18 + P39 and P39 ELISA to determine the seroprevalance of *Campylobacter* specific IgA and IgG antibodies in BD, AE, GBS, RA, and IBD respectively; we tested BD, AE, GBS, RA, and IBD sera for the presence of antibodies against *Helicobacter pylori* and *Yersinia enterocolitica* which are known to cross-react with *Campylobacter* antigens [24] and *Mycoplasma pneumonia* and *Borrelia afzelii* that cause similar clinical symptoms as those observed in campylobacteriosis associated post-infectious sequelae.

## Materials and methods

### Sera tested in the study

Sera tested in this study were collected from 91 GBS patients, 60 AE patients, 50 RA patients, 39 IBD patients and 80 BD.

The GBS cohort comprised of three sera from confirmed MFS patients and the remaining 88 were AMAN, AIDP and AMSAN suspected cases, which had not been clinically distinguished. Mean age of the patients was 61.2 ± 17.1 years; the age median was 66.0 ± 13.6 years and the proportion of male to female patients was 46.8 %:53.2 %. The sera of patients with acute diarrhea and a *Campylobacter-*positive stool culture were included as positive control. The mean age of AE patients was 47.5 ± 24.3 years, the age median was 51.0 ± 21.1 years, and the proportion of male to female patients was 59.3 %:40.7 %. The mean age of RA patients was 39.7 ± 22.0 years, the age median was 43.5 ± 19.5 years and the proportion of male to female patients was 48.0 %:52.0 %. The serum samples were collected at an acute flare-up stage of arthritis. The IBD patient group’s age mean was 37.3 ± 21.5 years, the age median was 38 ± 18.2 years, and the proportion of male to female patients was 40.7 %:59.3 %. The mean age of BDs was 39.2 ± 21.3 years, the age median was 42.2 ± 20.1 years, and the proportion of male to female patients was 48.4 %:52.6 %. BD sera were taken in July 2011 to serve as control group to estimate the seroprevalence of *Campylobacter*-specific antibodies in healthy individuals. All patients had been hospitalized in the University Medical Center Göttingen from 2001 to 2011 but there were no GBS cases in 2004 and 2005. All sera were stored at −80 °C until testing.

### Optimized ELISA assays

ELISA embedded with *C. jejuni* proteins P39 and OMP18 as antigens, singularly and combined, were prepared. Initially, the recombinant proteins P39 and OMP18 were expressed and purified as described before [22]. Then both proteins were diluted in bicarbonate buffer (pH = 8.4) to a final concentration of 3.0 μg/mL of P39 and 5.0 μg/mL of OMP18. NuncMaxiSorp® 96-well plates (Thermo Fisher Scientific Inc., Langenselbold, Germany) were coated with 100 μL protein solution of P39 or OMP18 or P39 + OMP18 at room temperature overnight in a wet chamber. After the coating procedure, the plates were washed three times with phosphate-buffered saline (PBS). Thereafter the plates were blocked with 1.0 % BSA in PBS for one hour at room temperature followed by lyophilization. After lyophilization the plates were stored dry. The measurement procedure was performed as described before [22], but with some modifications. First, the patient sera were used at 1:100 dilutions. The secondary horseradish peroxidase-labeled goat anti-human IgA and IgG antibodies were used at a dilution of 1:4000 (anti-human IgA) and 1:50,000 (anti-human IgG; KPL, Gaithersburg, USA). Therewith, we significantly increased the amount of antigen used and decreased the concentration of the secondary antibody in order to achieve higher sensitivity than previously described [22]. Signal intensities above the cut-off value 10.0 + 1.0 Virotec units (VU = 10*OD-sample/OD-Cut-Off) were considered as positive, below the cut-off value 10.0 – 1.0 VU were considered as negative, and in the range of cut-off value 10.0 +/− 1.0 VU were considered as borderline.

### MIKROGEN-recomLine *Campylobacter* blot and whole cell lysate-immunoblot

AE and GBS patient and BD sera were tested on MIKROGEN-recomLine *Campylobacter* IgA/IgG blot and whole cell lysate-immunoblot. Analysis using the well-established *recomLineCampylobacter* IgA and IgG blot (MIKROGEN Diagnostik, Neuried, Germany) was done as recommended by the manufacturer. On the other hand, the whole cell-lysate (WCL) line-blot was prepared and measurement carried out as previously described [23]. The evaluation of the sera on the whole cell lysate-immunoblot was determined by their responses to PEB1, PEB2, PEB3 and PEB4 antigens [23].

### *Helicobacter*, *Mycoplasma*, *Yersinia*, and *Borrelia* immunoblots

*Helicobacter pylori* LINE, *Mycoplasma pneumonia* LINE, *Yersinia enterocolitica* LINE, and *Borrelia* LINE Immunoblot assays (Sekisui Virotech GmbH, Rüsselsheim, Germany) were used for the detection of antibodies against *Helicobacter pylori*, *Mycoplasma pneumoniae*, *Yersinia enterocolitica*, and *Borrelia afzelii*, respectively. The prevalence of IgG and IgA antibodies against these pathogens was determined in all sera except against *B. afzelii* where only the prevalence of IgG was determined.

### Statistical analyses

The χ^2^-test was used to test for significant differences. The obtained p-values are indicated as ‘*’ (*p* > 0.05), ‘**’ (*p* < 0.05), or ‘***’ (*p* < 0.001) as shown in Table 2. Calculation of ROC curves and their comparison was performed using the ROC-Excel-Tool (ACOMED Statistik, Leipzig, Germany).

## Results

### Determination of OMP18, P39 and OMP18 + P39 ROC AUC values during detection of *Campylobacter* specific antibodies in BD, AE, GBS, RA and IBD

**Fig. 1:**
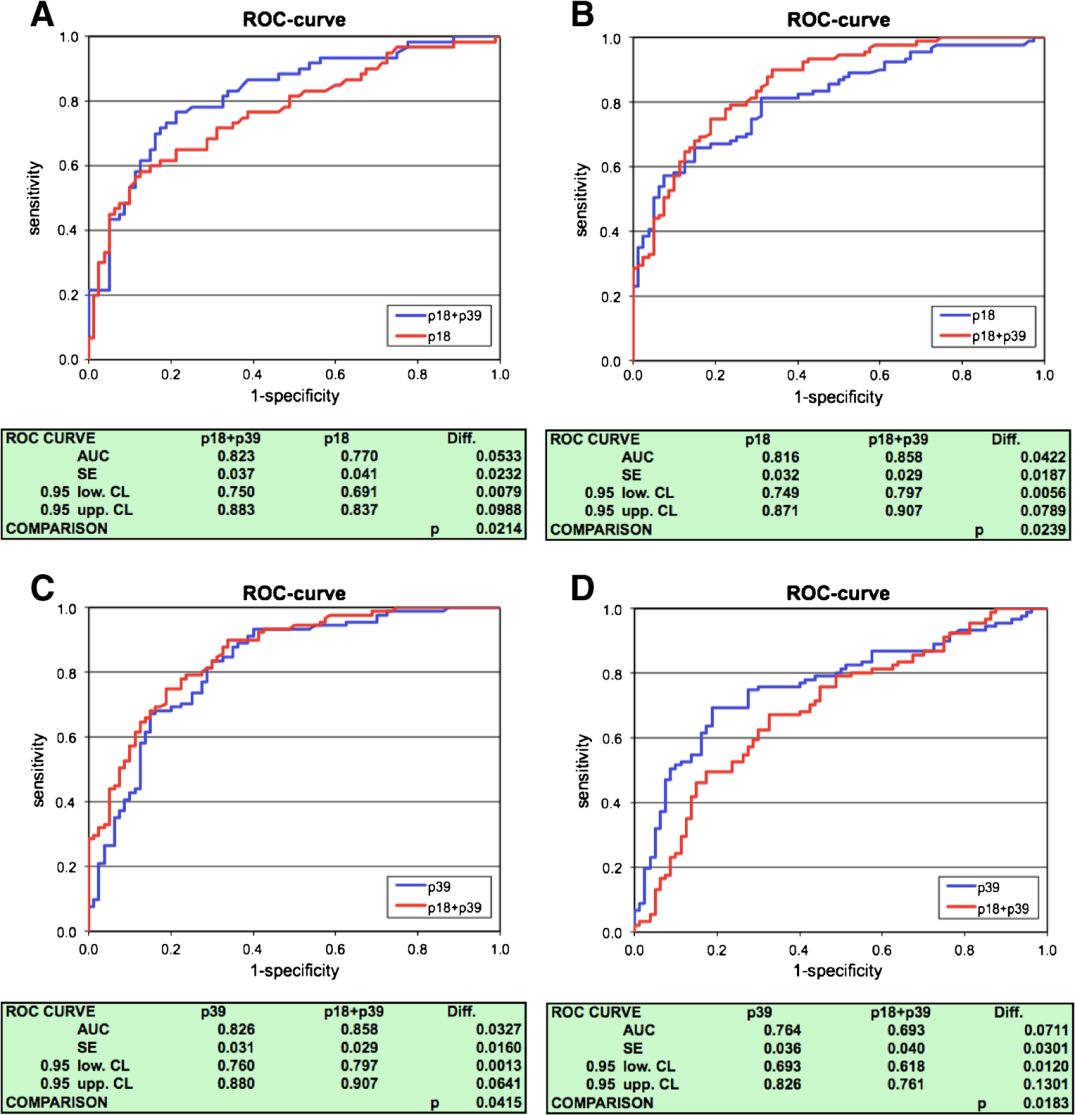
Receiver operating characteristic (ROC) curves comparing the antigens/antigen-combinations P18, P39, and P39 + P18 for the detection of anti-*Campylobacter* antibodies. **a** Sera of culture-positive acute *Campylobacter*-enteritis patients tested for anti-*Campylobacter* IgA. **b**, **c** Sera of GBS patients tested for anti-*Campylobacter* IgA. **d** GBS patients tested for anti-*Campylobacter* IgG. *AUC* area under the curve, *SE* standard error, 0.95 low./upp. *CL* 0.95 lower and upper confidence limits, *p* p-value

Evaluation of OMP18, P39, and OMP18 + P39 AUCs revealed that ELISA embedded with antigen combination OMP18 + P39 has a significant advantage in *Campylobacter*-specific IgA detection as compared to ELISA embedded with OMP18 in AE sera (*p* < 0.05; Fig. 1a), in GBS sera (*p* < 0.05; Fig. 1b) and to ELISA embedded with antigen P39 tested with GBS sera (*p* < 0.05; Fig. 1c). The comparison of AUCs for the detection of *Campylobacter*-specific IgG antibodies showed no significant differences between ELISAs embedded with OMP18, P39, and OMP18 + P39 except AUC of ELISA embedded with P39 which was significantly larger as compared to AUC of ELISA embedded with OMP18 + P39 (*p* < 0.05; Fig. 1d) testing GBS sera. ROC AUCs of antigens MOMP, PEB1, PEB2, and PEB4 and WCL were significantly smaller compared to antigens OMP18, P39 and OMP18 + P39 for both IgA and IgG (results not shown).

Table 1 lists sensitivity, specificity, positive predictive values (PPV) and negative predictive values (NPV) of tested individual antigens OMP18, P39, MOMP, PEB1, PEB2, and PEB4 as well as the combined antigens OMP18 + P39, and whole cell lysate estimated on sera of stool culture positive campylobacteriosis patients and healthy blood donors. With a specificity of approximately 90 % the OMP18, P39 and OMP18 + P39-based ELISAs showed a sensitivity of 57.1 %, 40.7 %, and 38.9 %, respectively, when tested for IgA and 51.9 %, 57.4 %, and 47.3 %, respectively, when tested for IgG. OMP18 and P39 tested on MIKROGEN recomLine blot demonstrated ≈ 10 % increase in specificity and ≈ 15 % decrease in sensitivity. In contrast, antigens MOMP, PEB1, PEB2, and PEB4 tested on MIKROGEN recomLine blot showed 0.0–18.3 % sensitivity when specificity of nearly 100 % was achieved. The selection of the cut-off is based on optical density raw data in a non-prametric approach. As shown by the calculated MIKROGEN evaluation index (Table 1), MOMP, PEB1, PEB2, and PEB4 resulted in 8 % increase in the specificity of IgA detection as compared to OMP18 + P39 ELISA. The sensitivity and specificity of IgG antibody detection was reduced by 7 % as compared to that of P39 ELISA. The WCL-based blot showed a sensitivity of 28.6 % and a specificity of 96.2 % in the detection of IgA antibodies and a sensitivity of 48.6 % and a specificity of 83.8 % in the detection of IgG antibodies.

**Table 1:** Sensitivity, specificity, positive (PPV) and negative predictive values (NPV) estimated on sera of stool culture positive campylobacteriosis patients and healthy blood donors

Antigen	Sensitivity [%]		Specificity [%]		PPV [%]		NPV [%|	
	IgA	IgG	IgA	IgG	IgA	IgG	IgA	IgG
OMP18 ELISA	57.1	51.9	90.5	90.8	82.1	79.4	73.6	73.4
P39 ELISA	40.7	57.4	90.9	90.5	75.9	81.6	68.6	74.4
OMP18 + P39 ELISA	38.9	47.3	91.0	90.8	75.0	78.8	68.3	70.4
MOMP MG	18.3	3.3	100	96.3	100	40.0	62.0	57.0
PEB4 MG	16.7	3.3	100	98.8	100	66.7	61.5	57.7
PEB2 MG	3.3	1.7	100	100	100	100	57.9	57.6
PEB1 MG	0.0	1.7	100	97.5	-	33.3	57.1	56.9
OMP18 MG	41.7	36.7	100	98.8	100	95.7	69.6	67.5
P39 MG	41.7	45.0	98.8	86.3	96.2	71.0	69.3	67.6
Index MG	56.7	50.0	98.8	83.8	97.1	69.8	75.2	69.1
WCL	28.6	48.6	96.2	83.8	76.9	58.6	75.0	77.2

*OMP18* outer membrane protein 18 kDa, *P39* protein 39 kDa, *MOMP* major outer membrane protein, *PEB1-4* perplasmic-binding proteins 1–4, *Index MG* numerical index for test evaluation that is the sum of the ascribed points of all positive antigens, i.e. bands with coloration more intense than the cut-off band, *WCL* whole cell lysate

These results show that a line blot or an ELISA based on antigen P39 and OMP18 have the highest sensitivity and specificity. Also, incorporation of antigens PEB1, PEB2, PEB4, and MOMP into line blot does not significantly improve sensitivity or specificity (Table 1).

### Evaluation of IgA, IgG and IgA/IgG reactivity in BD, AE, GBS, RA and IBD sera

OMP18 + P39-based ELISA for detection of antibody IgA and P39-based ELISA for detection of antibody IgG were used to determine the *Campylobacter* seroprevalence in BD, AE, GBS, RA and IBD sera.

As shown in Table 2, the reactivity of IgA antibodies in BD sera was 9–12 %, in culture-positive AE sera 35–45 % (*p* < 0.001), in GBS sera 48–55 % (*p* < 0.001), in RA sera 34–40 % (*p* < 0.05) and in IBD sera it was 26–31 % (*p* < 0.05). *Campylobacter*-specific IgA antibody prevalence was high in GBS followed by AE, RA and IBD sera in that order.

**Table 2:** Seroprevalence of *Campylobacter* (using P18, P39 & P18&P39), *Helicobacter pylori*, *Mycoplasma pneumoniae*, *Yersinia enterocolitica*, and *Borrelia afzelii* in the different patient groups

*Bacterium*		*Campylobacter* OMP18		*Campylobacter* P39		*Campylobacter* P18 + P39		*Helicobacter pylori*		*Mycoplasma pneumoniae*		*Yersinia enterocolitica*		*Borrelia afzelii*
Ig class		IgA	IgG	IgA	IgG	IgA	IgG	IgA	IgG	IgA	IgG	IgA	IgG	IgG
BD	+	9 % (7/80)	9 % (7/80)	9 % (7/80)	**9 % (7/80)**	**9 % (7/80)**	9 % (7/80)	9 % (7/80)	9 % (7/80)	9 % (7/80)	8 % (6/80)	9 % (7/80)	9 % (7/80)	9 % (7/80)
	±	8 % (6/80)	5 % (4/80)	4 % (3/80)	**8 % (6/80)**	**3 % (2/80)**	5 % (4/80)	4 % (3/80)	9 % (7/80)	1 % (1/80)	6 % (5/80)	5 % (4/80)	1 % (1/80)	6 % (5/80)
	-	84 % (67/80)	86 % (69/80)	88 % (70/80)	**84 % (67/80)**	**89 % (71/80)**	86 % (69/80)	88 % (70/80)	83 % (66/80)	90 % (72/80)	86 % (69/80)	86 % (69/80)	90 % (72/80)	85 % (68/80)
AE	+	53 %*** (32/60)	45 %*** (27/60)	37 %*** (22/60)	52 %*** (31/60)	35 %*** (21/60)	43 %*** (26/60)	3 %* (2/60)	2 %* (1/60)	3 %* (2/60)	25 %** (15/60)	37 %*** (22/60)	15 %* (9/60)	22 %** (13/60)
	±	7 % (4/60)	13 % (8/60)	10 % (6/60)	**10 % (6/60)**	**10 % (6/60)**	8 % (5/60)	7 % (4/60)	0 % (0/60)	3 % (2/60)	5 % (3/60)	12 % (7/60)	13 % (8/60)	8 % (5/60)
	-	40 % (24/60)	42 % (25/60)	53 % (32/60)	**38 % (23/60)**	**55 % (33/60)**	48 % (29/60)	90 % (54/60)	98 % (59/60)	93 % (56/60)	70 % (42/60)	52 % (31/60)	72 % (43/60)	70 % (42/60)
GBS	+	45 %*** (41/91)	26 %** (24/91)	35 %*** (32/91)	37 %*** (34/91)	48 %*** (44/91)	37 %*** (34/91)	19 %** (17/91)	10 %* (9/91)	10 %* (9/91)	21 %* (19/91)	41 %** (37/91)	20 %* (18/91)	38 %*** (35/91)
	±	9 % (8/91)	15 % (14/91)	10 % (9/91)	**16 % (15/91)**	**7 % (6/91)**	9 % (8/91)	7 % (6/91)	13 % (12/91)	4 % (4/91 %)	8 % (7/91)	18 % (16/91)	11 % (10/91)	9 % (8/91)
	-	46 % (42/91)	58 % (53/91)	55 % (50/91)	**46 % (42/91)**	**45 % (41/91)**	54 % (49/91)	75 % (68/91)	77 % (70/91)	86 % (78/91)	71 % (65/91)	42 % (38/91)	69 % (63/91)	53 % (48/91)
RA	+	40 %*** (20/50)	34 %** (17/50)	26 %** (13/50)	54 %*** (27/50)	34 %** (17/50)	30 %** (15/50)	8 %* (4/50)	8 %* (4/50)	6 %* (3/50)	36 %*** (18/50)	30 %** (15/50)	38 %*** (19/50)	8 %* (4/50)
	±	8 % (4/50)	14 % (7/50)	10 % (5/50)	**12 % (6/50)**	**6 % (3/50)**	18 % (9/50)	2 % (1/50)	0 % (0/50)	6 % (3/50)	8 % (4/50)	10 % (5/50)	14 % (7/50)	12 % (6/50)
	-	52 % (26/50)	52 % (26/50)	64 % (32/50)	**34 % (17/50)**	**60 % (30/50)**	52 % (26/50)	90 % (45/50)	92 % (46/50)	88 % (44/50)	56 % (28/50)	60 % (30/50)	48 % (24/50)	80 % (40/50)
IBD	+	33 %** (13/39)	18 %* (7/39)	26 %** (10/39)	44 %*** (17/39)	26 %** (10/39)	23 % * (9/39)	5 %* (2/39)	3 %* (1/39)	5 %* (2/39)	26 %** (10/39)	28 %** (11/39)	21 %* (8/39)	26 %** (10/39)
	±	5 % (2/39)	13 % (5/39)	3 % (1/39)	**3 % (1/39)**	**5 % (2/39)**	13 % (5/39)	18 % (7/39)	10 % (4/39)	8 % (3/39)	8 % (3/39)	15 % (6/39)	18 % (7/39)	3 % (1/39)
	-	62 % (24/39)	69 % (27/39)	72 % (28/39)	**54 % (21/39)**	**69 % (27/39)**	64 % (25/39)	77 % (30/39)	87 % (34/39)	87 % (34/39)	67 % (26/39)	56 % (22/39)	62 % (24/39)	72 % (28/39)

*AE* acute campylobacteriosis (reference: stool culture), *GBS* Guillain-Barré-Syndrome, *RA* reactive arthritis, *IBD* inflammatory bowel disease (Morbus Crohn & ulcerative colits); + positive, ± borderline, - negative.

Different significance levels in the comparison of a particular patient group with blood donors (BD) are indicated with ‘*’ (*p* > 0.05 = not significant), ‘**’ (*p* < 0.05 = significant), and ‘***’ (*p* < 0.001 = significant)

As shown in the same table, the P39-based prevalence of *Campylobacter*-specific IgG antibodies in BD was 9–17 %, in AE it was 52–62 %, in GBS 37–53 %, in RA 54–66 %, and in IBD it was 44–47 % (*p* < 0.001). *Campylobacter*-specific IgG antibody prevalence was high in RA sera followed by AE, IBD and GBS sera in that order.

Combined IgA/IgG reactivity was found to be 16–26 % in BD, 62–72 % in AE, 60–65 % in GBS, 70–78 % in RA and 49–56 % in IBD sera.

### Comparison of *H. pylori*, *M. pneumoniae*, *Y. enterocolitica*, and *B. afzelii* specific antibody prevalences in AE, GBS, RA, IBD and BD sera

There was no significant difference in *H. pylori*-specific antibody-prevalence in AE, RA, and IBD patient sera compared to BD sera. However, antibody-prevalence of *H. pylori*-specific IgA in GBS sera was significantly higher (Table 2); 9–17 % anti-*Campylobacter* and anti-*Helicobacter* antibody double positive sera were found in all sera (AE, GBS, RA, IBD, and BD sera). See Table 3 for a particular group.

**Table 3:** Percentages and absolute numbers of *Campylobacter*-, *Helicobacter pylori*-, *Mycoplasma pneumonia*-, *Yersinia enterocolitica*-, and *Borrelia afzelii*-positive tested (IgA and IgG) serum samples as well as percentages and absolute numbers of double positive tested serum samples in all 320 tested patients and blood donors

Bacterium	*Campylobacter* IgA^+^ *or* IgG^+^	*Helicobacter pylori* IgA^+^ *or* IgG^+^	*Mycoplasma pneumoniae* IgA^+^ *or* IgG^+^	*Yersinia enterocolitica* IgA^+^ *or* IgG^+^	*Borrelia afzelii* IgG^+^
***Campylobacter*** **IgA** ^**+**^ ***or*** **IgG** ^**+**^	50–57 % (159–183)	9–17 % (28–53)	16–23 % (51–75)	23–35 % (75–112)	13–22 % (42–70)
***Helicobacter pylori*** **IgA** ^**+**^ ***or*** **IgG** ^**+**^	9–17 % (28–53)	13–23 % (43–73)	4–11 % (13–34)	6–14 % (19–46)	5–11 % (15–35)
***Mycoplasma pneumoniae*** **IgA** ^**+**^ ***or*** **IgG** ^**+**^	16–23 % (51–75)	4–11 % (13–34)	25–34 % (80–108)	11–21 % (34–68)	6–13 % (18–40)
***Yersinia enterocolitica*** **IgA** ^**+**^ ***or*** **IgG** ^**+**^	23–35 % (75–112)	6–14 % (19–46)	11–21 % (34–68)	34–50 % (110–159)	11–19 % (35–61)
***Borrelia afzelii*** **IgG** ^**+**^	13–22 % (42–70)	5–11 % (15–35)	6–13 % (18–40)	11–19 % (35–61)	22–29 % (69–94)

Percentages are given in relation to all 320 serum samples included in this study. The absolute numbers of positive until positive plus borderline tested sera is given in brackets

There was no significant difference in reactivity of anti-*M. pneumonia* IgA-antibodies in all sera. But there was a significant increase (*p* < 0.05) in the reactivity of *M. pneumonia*-specific IgG in AE, RA, and IBD sera but not in GBS sera (Table 2); 16–23 % of all tested sera were positive for both anti-*Campylobacter* antibodies and anti-*M. pneumonia* antibodies (Table 3); 4 % of GBS sera that were negative for anti-*Campylobacter*-reactive antibodies showed both IgA and IgG antibodies reactive against *M. pneumonia*. The anti-*Campylobacter* antibody and anti-*M. pneumonia* antibody double-positive (IgA and IgG) rate was 23–33 % in GBS sera (results not shown).

The prevalence of *Y. enterocolitica*-reactive IgA antibodies significantly increased (*p* < 0.05) in GBS, RA, AE, and IBD sera. *Y. enterocolitica*-reactive IgG antibodies significantly increased (*p* < 0.001) in only RA sera. Also, 15–18 % (IgA), 8–11 % (IgG), and 23–35 % (IgA + IgG) of all tested sera were positive for both *Y. enterocolitica*- and *Campylobacter*-reactive antibodies (Table 3). Specifically, a significant increase of double-positive rate for IgA antibodies was 17–30 % in AE, 19–26 % in GBS and 18–26 % in RA sera. Increase of double-positive rate for IgG was 14–16 % in RA sera only. Anti-*Yersinia* antibodies (IgA/IgG) in *Campylobacter* antibody-negative sera were found in 3–18 %/3–5 % of AE, 12–19 %/7–9 % of GBS, 10–12 %/6–14 % of RA, and 5–10 %/8–13 % of IBD sera.

No significant difference in anti-*B. afzelii*-IgG antibody prevalence was observed between RA and BD sera. However, there was a significant increase (*p* < 0.05) in the prevalence of anti-*B. afzelii*-IgG antibodies in AE, GBS, and IBD patients; 13–22 % of all tested sera were anti-*Campylobacter* IgA and IgG antibody and anti-*Borrelia* IgG antibody double positive (Table 3). This double-positive rate was significantly (*p* < 0.05) above average in GBS patients (26–30 %) but below average in RA-patients (6 %) (results not shown).

## Discussion

In a generation of seroprevalence data, antigen or antigen combination is decisive for the test concerned. Generally, the reactivity of the immunoglobulin classes IgA and IgG is determined in *Campylobacter* serodiagnostics because they have been found to have high sensitivity [25]. Immunoglobulin class IgM is rarely used because of its low sensitivity [23, 25]. Routine serodiagnostics of prior *C. jejuni*-infections is performed using an ELISA or an immunoblot with whole cell lysate or recombinant antigens [20, 23, 25]. Recombinant antigens including MOMP [26, 27], OMP18 [28], P39 [22], Cj0069 [20], and PEB 1, PEB 2, PEB 3, and PEB 4 [29] are used to detect *Campylobacter* specific antibodies. However, the sensitivity of OMP18 and P39 in relation to MOMP, PEB1, PEB2, PEB3 and PEB4 is unknown.

Therefore, one of the objectives of this study was to determine which antigen between OMP18, P39 and antigen combination (OMP18 + P39) is the most sensitive and specific for detecting anti-*Campylobacter* antibodies in the diagnosis of acute campylobacteriosis and post-infectious sequelae. According to our data, an ELISA based on the combination of OMP18 + P39 shows the ROC AUC maximum for IgA antibodies whereas an ELISA based on P39 alone exhibits the maximal ROC AUC for IgG antibodies. Antigens P39 and OMP18 as part of the MIKROGEN recomLine blot show almost the same values for sensitivity, specificity, PPV and NPV as those of antigens P39 and OMP18 being part of an ELISA. Therefore, it is evident that a line blot or an ELISA based only on antigens P39 and OMP18 + P39 are the most sensitive and specific in detecting *Campylobacter*-specific antibodies in all patient groups. Additional detection of anti-MOMP, -PEB1, -PEB2, and -PEB4 immune reactivity does not significantly improve *Campylobacter* serology. Consequently, the testing of antibodies using these antigens in *Campylobacter* serology should be discouraged and testing of antibodies using P39 and OMP18 + P39 encouraged.

In this study 16–26 % of healthy BD tested positive for both *Campylobacter*-specific IgA and IgG antibodies. The serum samples of the healthy blood donors were collected during a summer month in which the *Campylobacter* prevalence is higher than in the winter months [30]. This might explain the increased prevalence rate of *Campylobacter*-specific antibodies in the healthy blood donor cohort in comparison to our previous studies [21, 22]. Similarly, 60–65 % of GBS patients tested positive for both *Campylobacter*-specific IgA and IgG antibodies; it therefore follows that 34–49 % (≈42 %) of GBS cases in this study are statistically associated with campylobacteriosis. Several studies have investigated the proportion of *Campylobacter*-triggered GBS and given an impression of an increase in *Campylobacter* associated GBS over time. For example, Winer et al. attributed 14 % [31], Rees et al. attributed 26 % [32], Jacobs et al. attributed 32 % [33], Hao et al. attributed 45 % [34] and our group attributed 80 % [21] of GBS cases to *Campylobacter*. This increase in the detection of seroprevalence can been attributed to improved detection methods. In this study, our data on German patients supports the findings of Hao et al. who associated 45 % of GBS cases with a prior *Campylobacter* infection in a study on a Japanese population [34] but varies from other studies which underestimated [31–33] or overestimated [21] the proportion of post-*Campylobacter* GBS. However, the varying proportion of AMAN, AMSAN and MFS cases that have been demonstrated to be associated with a prior *Campylobacter* infection relative to AIDP cases in GBS study populations [6] could be another reason for these differences in the prevalence of *Campylobacter*-specific antibodies. Future studies are required to address this thought.

Furthermore, we tested all GBS sera for *H. pylori-*, *M. pneumoniae-*, *Y. enterocolitica*, and *B. afzelii*-reactive antibodies. The relatively high rate of double *Mycoplasma*- and *Campylobacter*-positive sera of GBS patients (23–33 %) indicates that there could be additive effects triggering autoantibodies causing GBS by sequential (or simultaneous) infections with *M. pneumoniae* and *C. jejuni*. Otherwise, there could be significant test cross-reactivity between both bacterial species. The rate of 4 % of *Mycoplasma*-antibody positivity in *Campylobacter*-negative GBS-patients substantiates our data for *Campylobacter* seroprevalence in GBS patients, because it delivers valid data explaining the etiology of the remaining *Campylobacter*-negative GBS cases.

In contrast to that, the increased rate of double *Borrelia* and *Campylobacter* seropositive GBS patients (26–30 %) indicates an association between *Campylobacter*-triggered neuronal disease and neuroborreliosis either in antigenic interference (serodiagnostics) or even in etiology.

In the present study, we demonstrate a *Campylobacter* seroprevalence (IgA + IgG) rate of 70–78 % in RA patients. By subtracting the prevalence of *Campylobacter*-specific antibodies (16–26 %) of healthy BD, about 44–62 % (≈53 %) of RA cases are associable with *Campylobacter*. Moreover, tests to determine *H. pylori-*, *M. pneumoniae-*, *Y. enterocolitica-*, and *B. afzelii*-reactive antibodies in all the sera showed higher *Campylobacter*-specific antibodies than those of other pathogens. The association of yersiniosis and RA is well described [35, 36] and reconfirmed by the seroprevalence data of this study. The relatively low *B. afzelii* seroprevalence rate (8–20 %) as well as the low rate of double *Borrelia* and *Campylobacter* antibody-positive sera in RA patients (6 %) indicates that the subgroup of *Borrelia*-reactive arthritis interferes only to a minor degree with post-*Campylobacter* RA.

One debatable question is the contribution of *Campylobacter* caused episodes of acute gastroenteritis to the pathogenesis of IBD. Recent investigations in gnotobiotic mice demonstrated that the composition of the intestinal flora plays a pivotal role in the pathogenesis of acute campylobacteriosis. Due to their microbiota composition, mice display a natural colonization resistance against *C. jejuni* [16, 17]. Quantitative analysis of the bacterial gut flora composition revealed two- to three-fold increased *Escherichia coli* loads in intestinal ‘humanized’ mice that are susceptible to *C. jejuni* compared to resistant mice recolonized with a murine gut flora [16, 17]. Likewise, high-elevated *E. coli* amounts increase the susceptibility to *S. enterica*-caused enteritis in mice [37–39]. Here a vicious circle starts because intestinal inflammation is associated with remarkable changes in the gut microbiota. Intestinal inflammation results in a decrease in the diversity of natural bacterial species in the gut leading to overgrowth of commensals such as *E. coli*, *Bacteroides* spp., and *Prevotella* spp. [40–44]. This dysbiosis in turn increases the susceptibility for *C. jejuni*- and *S. enterica*-caused enteritis. Additionally it was shown that *E. coli* LPS triggers TLR-4-signalling, which is a key signal for initiation and continuation of enteritis [41, 42]. Continuous inflammation is one pivotal parameter in the multifactorial pathogenesis of IBD [13, 45]. In this regard we are faced with a delicate codependency situation. On one hand, IBD patients are more susceptible to enteric pathogens [13, 45, 46], while on the other hand, recurrent episodes of acute bacterial enterocolitis trigger the manifestation of IBD in susceptible individuals [11, 12, 47]. Thus, the significantly (*p* < 0.001) increased *Campylobacter*-seroprevalence in IBD patients, which ranges from 23 to 40 % (≈32 %), subtracting the prevalence of *Campylobacter*-specific antibodies in 16–26 % of healthy individuals (BD) in our study, supports the theory of campylobacteriosis-triggerd IBD as well as the theory of an increased susceptibility for *Campylobacter* in IBD patients. Recent studies performed on rodent mice have revealed that *H. hepaticus* and *H. bilis* employ the same mechanism to trigger IBD [48]. Therefore, further studies are required to determine if a similar situation applies to other enteric pathogens.

The overall low *H. pylori*-seroprevalence rate (13–23 %) and especially the low rate of double *Helicobacter*- *and Campylobacter*-reactive sera (9–17 %) indicates that there is very little influence of cross-reactivity between both bacterial species in this study. The reasonably high rate of *Yersinia*-antibody positive sera especially among patients who were also tested *Campylobacter*-seropositive may be explained by unspecific (re-) activation of the specific intestinal immune response against an intestinal pathogen (especially in IBD) but also by antigenic interference, that would be a significant weakness in *Campylobacter* serodiagnostics. Therefore, this aspect has to be further addressed in future studies.

In conclusion, we show that antigens OMP18 + P39 (for IgA) and P39 (for IgG) have larger ROC AUCs than WCL, OMP18, MOMP, PEB1, PEB2 and PEB4 antigens. Therefore, their usage in diagnostics of previous *Campylobacter* infections will produce much more reliable results. In addition, we present valid data showing that a higher proportion of post-infectious sequelae, namely, GBS, RA and IBD are triggered by *Campylobacter* spp. as compared to *H. pylori*, *M. pneumoniae*, *Y. enterocolitica*, and *Borellia* spp. However, there is evidence for codependency between *Campylobacter* infections and infections with these pathogens to trigger particular post infectious sequelae. Therefore, further studies should address how the sum of antibodies specific for these pathogens increases the risk to trigger a particular post infectious sequel and to which extent cross-reactivity of these antibodies affects the specificity of diagnostic tests.
